# Valedictory Address

**Published:** 1848-07

**Authors:** James Taylor


VALEDICTORY ADDRESS, *delivered to the Graduating Class of the Ohio College of Dental Surgery, at the annual commencement, February 20th*, 1848.

YB JAMES TAYLOR, M. D. D. D. S.
*Prof. of Practical Dentistry and Pharmacy, &c.—Dean of Faculty.**

GENTLEMEN:

Custom renders it necessary that I, as Dean of the Faculty, should on this occasion make a few remarks. I am glad to think that a long address will not be expected. I feel, however, that I am too well recovered from my long illness of the winter, to offer ill health as an excuse for not addressing you at all. I hope, however, inasmuch as the duties of my chair have pressed unusually upon me during the latter part of the session, that no other apology will be necessary for the brevity of my remarks for the present.

* We have been repeatedly (by different members of the Profession as well as by members of the Class) requested to publish in the Register the short Valedictory Address we delivered on the occasion of the last commencement. We have deferred this to the present, having, as we supposed, more important and interesting matter with which to fill our pages. Did we not consider the remarks we made on that occasion equally applicable to the Profession at large as to the class more particularly addressed, we should still have refused to let it appear in the Register. J. T.

It is now too late to fall back on the teachings of the winter, and give you a long lecture on aught which may pertain to the particular branch of Dental Science it is my duty to teach. If our time has been misspent, vain regrets are of no avail—time lags not, stops not, makes no shifts or turns to suit our ease or leisure. The most, then, that we can do, is to keep up in future with her onward progress—let each day—each hour—yea, minute—be improved; in this way alone can we make our pursuit of knowledge keep apace with her rapid flight.

How few can we find who, on a retrospect of the past, but will make resolves that the future shall be better improved? Yet they live on in the same dull, monotonous, inefficient way.

Gentlemen, you will please recollect that it is one thing to resolve, but to do is different. Give me the man who does as he resolves—whose resolution scarce precedes the effort to accomplish—"whose *iron* will" bears no parly with wishes and feelings which cry another day. Take now our case, to-morrow we will do thus and thus.

He who would do aught noble or worthy of imitation must work physically or mentally—high attainment in any science or pursuit of life, is not oft, if ever attained by the sluggard, who cries—"a little more sleep, a little more slumber, a little more closing of the eye-lids together;" but he who expects to attain the object of his pursuit or aspirations must be vigilant, active and persevering. Monterey yielded not at the first thundering of our cannon. Chapultepec and the once splendid and beautiful city of the Montezumas was not at once taken by assault—no, but the continued action and unyielding *will* of our officers and soldiers led them on to victory.

How gradual has been the acquisition of that knowledge we possess: here a mite and there a mite has been added to the general stock; no sudden illumination has been poured upon us; but one fact after another has been worked out, and, as it were, disentangled from the error which so oft at first appeared to surround and envelope the truth. In our Pathological investigations, this appears to be peculiarly the case. A prominent fact is presented for our consideration, but it only discloses the outer covering, the mere shell, which contains much which is hidden and mysterious to our view. The beautiful machinery of our system in its physiological relations, have been, 'tis true, so elucidated by observation, verified by repeated demonstrations, that the natural and healthy operation of each part is, to a great extent, made apparent to our senses. Yet how often are these operations disturbed, and instead of affording to us ease and comfort, every movement is attended with pain. Some mysterious and, for the time, all-pervading power works within, and plants disease in our system. To subdue and control this, Gentlemen, at least in one department of medical science, is your province. You have made this your study, and the period long looked for when you should be prepared to receive the honors of this Institution, and go forth to discharge the duty of a practitioner, has arrived. Let me congratulate you on this event; at the same time I would impress on your minds the fact that this is an important era in your life—yes! I will repeat, an important *period* of your life—for you are now where hundreds, yea, thousands, make shipwreck of their future usefulness—where thousands fail to fulfil or carry out the just expectations which fond friends may have formed concerning their onward and upward career in life—upward, because there is such a thing as an elevation in life which but few attain. The scale indeed turns both ways; and on this there is only three positions you can occupy.—You may remain at the centre, the great broad fulcrum on which the scale of life is balanced, or you may take the end of the beam which ascends or descends. Let me, however, impress this truth upon your minds, which is, that the sluggard's own weight will drag him down—while it will require your continued effort to move upward on the end which ascends.

Think not, gentlemen, that the act this evening just consummated gives you, in and of itself, the acme of dental knowledge. True, 'tis a certificate of qualifications, and should always be of the highest commendation. It implies study, and shows that you have, on your part, embraced the best possible opportunities for preparation in that department of science you expect to pursue in life. So far then, you have pursued an honorable, conscientious, and high-minded course, and the award which merit demands has been conferred. But, gentlemen, think not all is obtained; but remember it is your duty to investigate for yourselves. Much which you have learned has, of necessity, by you not yet been demonstrated, but is the result of the investigations and observations of others. Much, 'tis true, has been already demonstrated, and so far as done to your satisfaction should be received; yet much also yet remains for you to test. To do this aright, continued study, continued labor will be necessary. You are now on that broad platform of dental science from which you should look out with a scrutinizing eye, and examine with care every branch of general science which may throw light on aught which pertains to your profession.

One great error with perhaps a majority of those who practice Dentistry is, that they expect to get out of two or three dental works all that is necessary, or that can in any way contribute to their success in practice. They limit their services to simply a few operations on the teeth; and the mere manipulations requisite to their performance is all they desire to aim at. With these views, and impelled by no higher motive, with no aspirations to excel, with nothing to govern their actions but sordid pelf, how can they succeed? They never can succeed, and never should be permitted to operate. True, they may work upon the credulous, and draw from their pockets a subsistence greater than from that pursuit in which they had been engaged. But who can count the injury they inflict, not only on those for whom they operate, but an injury, a lasting disgrace on the profession itself.

Your studies, gentlemen, have been such, that a wide field of scientific research is spread out before you—the foundation has been laid for a beautiful and enduring superstructure, one which, if reared aright, shall reflect lasting credit on the builder, as well as upon your alma mater. Will not, then, your time, your talents, and your energy be applied for this great object. *Great,* because it carries out the noblest aspirations of our nature, fits us for usefulness in high life, and carries with it a rich reward in the knowledge we obtain. The old maxim, that "time is money," should never be lost sight of. Were we to feel the full force of this, and constantly endeavor to use it as our money should be used, how seldom would we waste the precious moments allotted unto us. 'Tis easy to look back and see much time that has been thrown away. Were it easy to gather it up again, how busy would many of us appear. Every student, I might add every man who wants system in study or labor, fails to accomplish much, or if not he gains his object by twice the toil he would otherwise do. Our studies, therefore, as well as the time for those studies, should be systematized. A habit is thus formed which renders study less irksome, and indeed makes it a pleasure rather than labor.

Gentlemen, thus far I have directed you to a high and noble aim, even your onward acqusition of true knowledge—that knowledge which shall give you, through God's blessing, power over disease, and a just and proper influence in society at large. Let me, then, urge you onward and upward; and may every step on the ladder which you ascend open wider and wider views of the wisdom and power of Him who has arranged in such beautiful order the vast storehouse from which your knowledge must be obtained.

Before I close my brief remarks, permit me to say a few words in regard to the duties you are about to assume. It was at one time my intention to have addressed you specially on this subject alone; but on reflection I thought it as well to point to a duty equally as important, and the advancement in which brings about almost certainly, the fulfilment of the other. He who makes every effort in his power to advance in the profession, is most generally prepared to discharge its duties aright. It leads also to a keen perception of those duties, and so long as he makes his profession the child of his love, you will always find him zealous of its right, and prepared, so far as his abilities will permit, to perform his duty.

What are the special duties of the Dentist? I answer, first, that to which I have called your attention, viz.: the most perfect and comprehensive knowledge of the profession which can be obtained. This might, perhaps, be called the duty of the student, *and it is his* special duty; but it ends not with his graduation, but extends through life, and rests as heavy upon him in practice as before; and I pity the man who, in his arrogance, places himself above being taught.

The second duty is to render all our services in that manner which shall most conduce to the good of our patient. Here, let me remark, that many obstacles will present, by which the inexperienced operator may be overruled and misled. The dictation of your patient should never, when contrary to your just views of the right course to be adopted, have the slightest influence. Study well the effect of your operation—the influence it may exert on the general system—the probable result as it regards durability and service—and after thus determining what is best, thus operate or decline your services. An early adoption of this rule will save you incalculable trouble, and perhaps no small disreputation. What is more ridiculous, than for an individual to employ a physician and prescribe for himself? A course of this kind would soon make nurses and apothecaries of our doctors, but would never advance the cause of medical science.

The third duty of the Dentist, viz.: that of selecting the best of materials for his use, is indeed embraced in that which has been said on the second, for without these we cannot operate "for the best good of our patient." But this is so important that we shall say a few words on this particular head. Experience has proven that *base* alloys in the mouth will exert a pernicious influence on the health of the general system, as well as on the teeth.

*Cheap Dentistry*, invariably and of necessity, induces the employment of the cheapest material, and the more alloy added to gold the cheaper it can be afforded. The intrinsic value of pure gold cannot go below the national standard (by weight), and when I see members (*so called*) of the profession offering their services for about the cost of good material, I must take it for granted that the profit comes from a deficiency in the quality and quantity of the article used. This you may take as certain, that good operations are as cheap in Cincinnati as they can *now* or *ever* will be, until we, as the Spartans, make gold of less value than iron. Let merit, gentlemen, and not cheap operations, get you practice. When thus obtained it is enduring, and affords to the conscience no sting to lash you in your waking or sleeping moments.

Many of the operations you are called upon to perform are of a painful and distressing kind—many of your patients will be morose and irritable, and should receive all the sympathy and courage you can give; firmness, but not roughness, will oft aid you much in your labor. Truth, and truth alone, on all occasions, (even with the child on whom you are about to inflict pain,) should be inviolate. How oft have I seen its violation bring trouble, again and again, on the parent—may I add, also, *trouble* to the Dentist?

Gentlemen, much, very much, might be said of the duty of the Dentist, and the importance of the profession; time, however, will not permit a more full elucidation of this subject. We are about to part, and that connection which has existed between us as teacher and student is about to be dissolved. That you will find in the life before you many warm friends—those who will sympathise with you in affliction, encourage you in difficulties, and sustain you in all your efforts to do good, I have no doubt; and since it is the lot of frail humanity to suffer from dental disease, I hope that your skill in affording relief will soon give you ample employ.

